# Determining the effect of tele-rehabilitation on patients with stutter using the goal attainment scaling (GAS)

**DOI:** 10.1186/s12911-021-01642-3

**Published:** 2021-10-12

**Authors:** Maryam Eslami Jahromi, Leila Ahmadian

**Affiliations:** 1 Department of Health Information Management, School of Health Management and Information Sciences, Iran University of Medical Sciences, Tehran, Iran; 2 Medical Informatics Research Center, Institute for Futures Studies in Health, Kerman University of Medical Sciences, Haft-bagh Highway, PO Box: 7616913555, Kerman, Iran

**Keywords:** Tele-rehabilitation, Speech therapy, Stuttering, Goal attainment scaling, Video conferencing

## Abstract

**Background:**

Lack of proper and timely patients' access to speech pathologists can affect the treatment and follow-up process; therefore, patients do not achieve the expected therapeutic goals. The aim of this study was to determine the effect of tele-rehabilitation on the stuttering patients using the goal attainment scaling (GAS).

**Methods:**

This interventional study was carried out on patients who visited the rehabilitation centers affiliated to the Jahrom Welfare Office. They underwent remote speech therapy using Skype. To evaluate the treatment outcomes of the stuttering patients, GAS was used.

**Results:**

The participants' speech and lingual skills improved using videoconferencing. The mean score of total GAS for patients was 53.08. Of 112 health goals, patients reached the expected or higher than expected levels in 78 goals.

**Conclusions:**

Rehabilitation through video conferencing was effective for patients with stuttering, improved their speech, and decreased their stuttering. Since, application of remote rehabilitation services can provide regular access to healthcare services, it can lead to improving patient treatment provide more frequent and faster treatment follow-up.

## Background

Telerehabilitation is a new and developing trend in the field of telehealth. It provides telecommunication rehabilitation services using communication technologies. The main reason behind the development of telerehabilitation is the provision of fair access to treatment for all individuals who live far away from health centers or for individuals with physical disabilities who cannot travel easily to such centers [1].

For the nations with vast and large population size such as Iran, many people have to travel a long distance to the nearest towns or cities to receive speech therapy services. However, telerehabilitation solved this problem by limiting the traveling time, and reducing the patient's fatigue [2].

Studies on speech-language rehabilitation by videoconferencing showed that patients improved with this method of therapy [3–7]. In order to implement remote speech therapy in Iran, given the specific conditions and available facilities in this country, the effectiveness and the achievement of the treatment goal of such services should be assessed before designing this therapy method. In our previous study, the patients’ satisfaction regarding applied infrastructure used to provide tele-speech therapy was assessed and the results showed that patients were satisfied [8].

Other studies done in other countries measured the therapeutic efficacy by calculating the percentage of stuttered syllables (SS%) [3, 9–14]. In the present study, goal attainment scaling (GAS) was used, because this scale overcame the weakness of traditional scales. It examines a set of standard questions regardless of the specific problems related to each patient and has higher accuracy than traditional scales. Furthermore, GAS has a positive therapeutic value, since it encourages patients to achieve their goals. Other advantages of GAS include having an easy application, considering individual goals set by each patient without taking other issues into account, assessing multiple domains, providing a total score of patient's performance improvement, and rendering measurable and applicable services in different situations of varying severity [15].

In the current study, a number of therapeutic goals were set for patients with stutterers to investigate whether remote speech therapy intervention can help patients to reach their intended goals. Therefore, the aim of this study was to determine the effect of telerehabilitation on patients with stutterers using the GAS.

## Methods

The present interventional study aimed at determining the effect of telerehabilitation on patients with stutters using remote speech therapy interventions.

### Ethics

This research was approved by the ethics committee of Kerman University of Medical Sciences (IR code: IR.kmu.REC. 1396 1085) in 2018.

The researcher asked the participants to complete the written informed consent forms. Moreover, they were also ensured about the confidentiality of their personal information. For patients younger than 16 year old, parents or legal guardians gave their consents. The study objective was explained to this group of patients in presence of their parents and when both patients and parents were agreed on the participation in the study, the form was signed by the parents. We confirm that all methods were carried out in accordance with the relevant guidelines and regulations.

### Participants

To carry out this study, individuals who visited the rehabilitation centers affiliated to the Jahrom Welfare Office were studied. In total, two rehabilitation centers Fatemeh al-Zahra and Haj Shekarriz are affiliated to Jahrom Welfare Office, which were selected to conduct the study. The participants were recruited among the individuals who visited these two centers in a 3-month period. Due to the limited number of stuttering adolescents who visited these two centers, the census method was applied and no sampling was conducted and all 30 patients entered the study (17 male and 13 female). The inclusion criteria for patients were having 14 years of age and higher, having stuttering disorders, referring to the mentioned rehabilitation centers, and having consent to participate in the study. The exclusion criteria included having less than 14 years of age, stuttering frequency of less than two percent from SSs, having lack of consent to participate in the study, having major diseases or underlying disorders that require a referral, and having previous speech rehabilitation over the past 6 months. All participants had developmental stuttering and stuttered from childhood.

### Treatment program

The Camperdown program treatment method was used in this study, which is a speech rehabilitation program for stuttering adults. This program consists of four stages of teaching treatment components, creating non-stuttering speech in the treatment environment, transmitting normal speech without a stutter in everyday situations, and reaching the maintenance stage [16, 17].

### Requirements of the technology

Remote speech therapy sessions were conducted for patients using videoconferencing. Video conferencing was established using Skype software. To conduct the therapy sessions, all participants used the same system and specifications during the study period. The appliances used for distant treatment included a laptop screen with a display resolution of 1366 × 768 pixels, Sony headset model MDR ZX110, and the Internet bandwidth of 1.99 megabytes per second.

### Evaluation technique

To evaluate and analyze the results of post-intervention treatment, GAS was used, which is a method for scoring the patient's predetermined goals. In this approach, each patient has a unique assessment concerning his/her therapeutic outcome, but scoring is done in a standard way. When applying GAS, the goal items are determined individually for each patient, and the expected levels of treatment options are determined. These levels are defined with the help of patients; they specify their prioritizations to guide the results of the intervention and to help the effectiveness of rehabilitation services [18].

The GAS process consists of 5 steps:

- *Step 1* Defining the expected goals

The goals are determined by interviewing with patients to identify the main areas of their problem, reach an agreement on the priorities of goals, and determine a specific deadline for the acquisition of goals. Therefore, the speech therapist determined specific therapeutic goals based on the speech problem and disability of each patient. In this study, a total of eight categories of therapeutic goals were specified; reducing avoidance behaviors, improving stuttering and physical symptoms, improving life quality, reducing fear of communicating, changing attitudes toward stuttering and communication, reducing fear of speaking, and increasing the effectiveness of communication. These goals were set for each patient individually according to his/her problems and therapeutic goals.

- *Step 2* Weighting the goals

Speech therapists, with the help of patients, scored the goals in terms of importance and difficulty. As a result, the final weight of each goal was calculated after multiplying the difficulty by the importance attributed to each goal. In the case that the weighting process was not used for goals, they were weighted as 1. The goal's importance and difficulty were scored as 1 when the goal had very little importance and difficulty, 2 when the importance and difficulty of the goal were at the moderate level, and 3 when the goal was very important and difficult [19]. In this study, the importance of goals was weighted from 1 to 3 for each patient.

- *Step 3* Goal scoring

One of the important features of GAS is setting some criteria for each patient to reach a successful outcome. These criteria were determined by the patient and speech therapist before the intervention. Then, each goal was scored on a 5-point Likert scale before the intervention. The speech therapist determined the levels, which should be as tangible and objective as possible. In the case that the patients rea the expected level, a score of zero was dedicated to them. If they earned a result of higher than the expected level, the score was + 1 (more than expected) or + 2 (much higher than the expected level). In the case that patients received a result of less than the expected level, the scores was − 1 (less than the expected level) or − 2 (much lower than the expected level) [18].

- *Step 4* Assessing the goal attainment

The treatment level obtained after the course of treatment is evaluated by the speech therapist [20, 21]. In this study, after completion of the therapeutic sessions, the level of patients' goal attainment was evaluated against the therapeutic purposes by the second therapist. In order to reduce the intervention bias and to have a better evaluation of the results, the second speech therapist was not aware of the treatment type received by the patients.

- *Step 5* Goal attainment scoring

To evaluate the effectiveness of treatment, the total score is used with regard to goal attainment using the T Score formula [22].

In the case that the results exceed the expectation level, the average score will be higher than 50. A useful quality check for GAS is considered 50 in the GAS practical guide developed by Turner–Stokes [22].

$${\text{Overall gas}} = 50 + \frac{{10\sum {{\text{W}}_{{\text{i}}} {\text{X}}_{{\text{j}}} } }}{{\left[ {\left( {1 - {\text{p}}} \right)\sum {{\text{W}}_{{\text{i}}}^{2} } + {\text{p}}\left( {\sum {{\text{W}}_{{\text{i}}} } } \right)^{2} } \right]^{1/2} }}$$

where W_i_ = the weight assigned to the i-th goal (if weight is equal, W_i_ = 1); X_i_ = the value obtained from the target rank (between − 2 and + 2); p = correlation of the expected goal's scales.

According to Kirusek and Sherman [18] p is often estimated as 0.3 for practical purposes; so, the simple equation can be:

$${\text{Overall gas}} = 50 + \frac{{10\sum {\left( {{\text{W}}_{{\text{i}}} {\text{X}}_{{\text{j}}} } \right)} }}{{\surd \left( {0.7\sum {{\text{W}}_{{\text{i}}}^{2} } + 0.3\left( {\sum {{\text{W}}_{{\text{i}}} } } \right)^{2} } \right)}}$$

### Measuring the stuttering severity

To measure stuttering severity, before starting the intervention and after completion of the treatment sessions, a stuttering severity instrument (SSI-4) was used. In order to investigate a change in the stuttering severity, the pre-test and post-test scores were compared using the paired samples t-test. More details can be found in [23].

## Results

All patients above 14 years old who visited the two centers (100%) received the remote speech therapy. The study included 56.7% male. The participants had the mean age of 23.23 ± 6.39 years old. More than half of the study population had non-academic education (56.6%) and most participants with university education had bachelor's degree (36.7%) (Table 1).

**Table 1:** The participants' demographic information

Demographic information	Frequency	Percent
Gender		
Male	17	56.7
Female	13	43.3
Age		
10–20	12	40
21–30	14	46.6
31–40	4	13.3
Education		
Middle school	7	23.3
High school diploma	10	33.3
Associate degree	1	3.3
Bachelor degree	11	36.7
Masters	1	3.3

Table 2 shows the participants' GAS scores after receiving remote speech therapy. A total of 112 therapeutic goal were defined. From these goals, 19 goals were related to reducing the avoidance behaviors, 30 goals were about reducing stutters, 19 goals were attributed to reducing physical symptoms, 18 goals were considered for improving the quality of life, 12 goals were set for reducing fear of communication, 11 goals were targeted at changing attitudes toward stuttering and communication, two were aimed at reducing fear of speaking, and one goal was to improve the effectiveness of communication and speech.

**Table 2:** The participants' GAS

Participants	GAS 1*	GAS 2	GAS 3	GAS 4	GAS 5	GAS 6	GAS 7	GAS 8	GAS T-score
1	− 1	0	†						42.63
2	0	+ 1	+ 2	+ 1					62.59
3		− 1		− 1	− 1		− 1		36.03
4		0		0					50
5		− 1	− 1		− 2			− 2	29.53
6	− 1	0		− 1					43.38
7		+ 2		+ 1			+ 1		65.45
8	+ 1	+ 2	+ 1	+ 2					81.05
9		− 1		− 2					35.43
10	− 2	0			− 2				31.61
11	0	+ 1	− 1		− 1	− 1			43.12
12	− 1	+ 1	− 1		+ 1	0			50.64
13	− 1	+ 2	0						61.03
14		− 1	− 2	− 2		− 1			29.53
15		0	− 2						40.18
16	+ 2	+ 2		+ 1	+ 2	+ 1			73.63
17		+ 2		+ 2		+ 2			75.7
18	0	+ 1	− 1		+ 2				58.01
19		0		0		0			50
20	+ 2	+ 2	+ 2	+ 2					77.94
21	+ 1	+ 2	+ 2						73.3
22	0	0	+ 1	0	+ 1				58.49
23	+ 1	0	+ 1	+ 1	0	+ 2			60.91
24	+ 1	0			0				52.57
25		− 1	+ 1	− 1					43.2
26	0	+ 1	0			+ 1			57.2
27	+ 1	+ 2	0	0		+ 2			68.86
28		0	− 1	0	0	− 1			45.96
29	+ 1	0	− 1		0				50
30	− 1	− 1	+ 1	0		0			44.61
Total									1592.58
Average									53.08

^*^GAS 1: Reduction of avoidance behaviors, GAS 2: Decrease of stutters, GAS 3: Reduction of physical symptoms, GAS 4: Improvement of the quality of life, GAS 5: Reduction of communication frustration, GAS 6: Change of attitudes towards stuttering and communication

^†^Empty houses: Therapeutic goals were not applied for the participant

In total, the patients reached the expected or higher levels of outcome in 78 goals (69.6%). The patients reached the expected levels in 31 goals (i.e., GAS score = 0). They also over passed the expected levels in 45 goals (i.e., the GAS score of 1+ or + 2). Of 30 patients, 26 (86.6%) reached the expected or higher level for at least one goal and 14 patients (46.6%) achieved all their therapeutic goals (at the expected or higher levels).

**Fig. 1:**
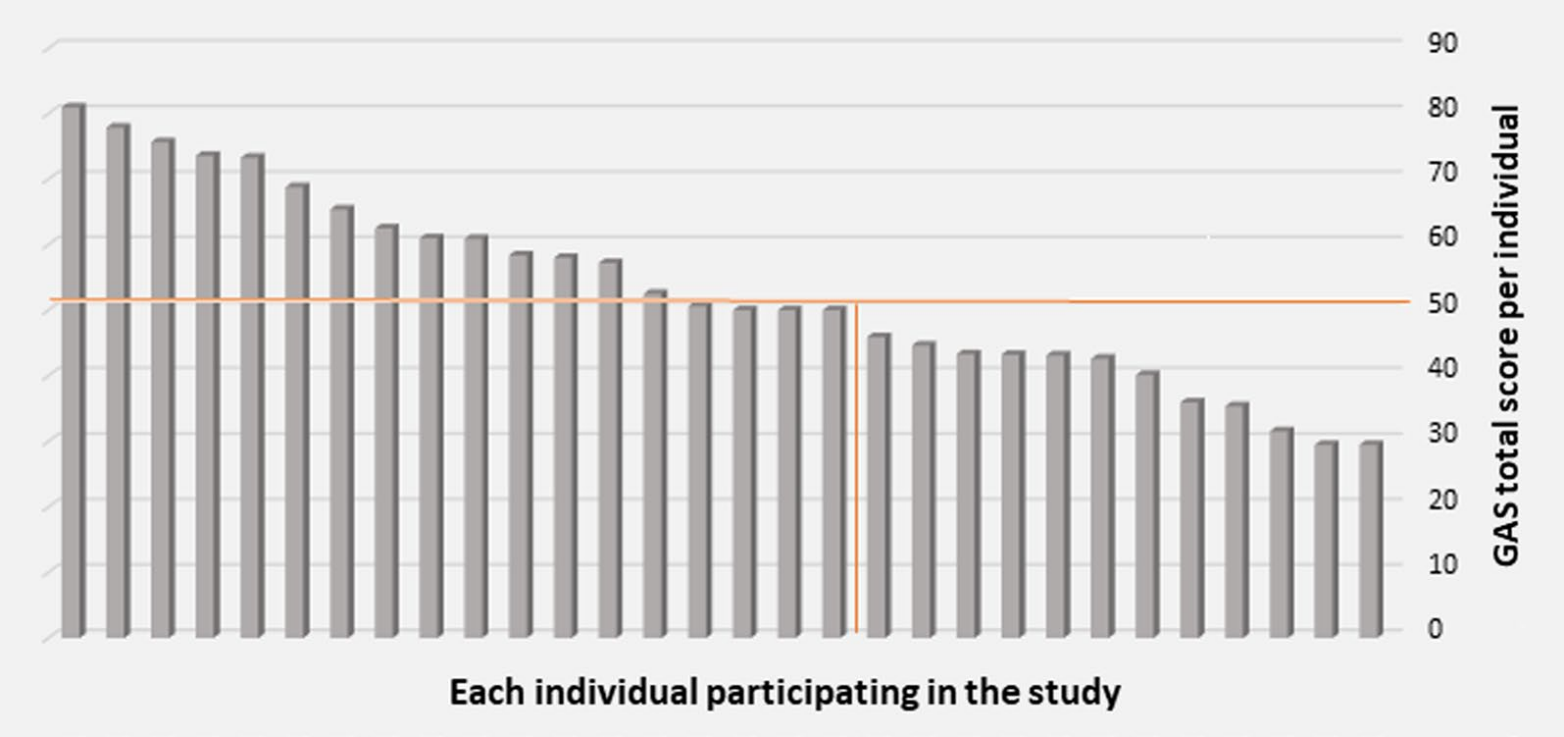
The Participants' GAS total score

The total GAS scores of patients were in the range of 29.53–81.05, with the mean of 53.08. The total GAS score of three patients (10%) was 50. The total GAS score of 15 patients (50%) was higher than 50, and the total GAS score of 12 patients (40%) was lower than 50. Therefore, 60% of patients reached the expected or higher levels of outcome after 15 treatment sessions of 45 min (Fig. 1).

The results of comparing stuttering severity showed that the average stuttering score of the patients significantly decreased from 27.9 in the pre-test to 14.10 in the post-test (*p* < 0.001).

## Discussion

The results of the present study showed that the participants' speaking and linguistic skills improved by videoconferencing and more than half of them reached the expected or higher levels of the defined goals.

In line with our findings, the results of the study by Fairweather et al. [6] on the effectiveness of a speech-language pathology program for students in New South Wales, Australia showed that the children's GAS mean scores were improved and most of the children) reached the expected levels or exceeded them. The results of the pediatric treatment confirmed that the provision of telemedicine was possible and acceptable. Lincoln et al. [7] also studied the feasibility and acceptability of the remote speech therapy pathology program from the viewpoints of patients. They concluded that the provision of remote speech therapy services was very acceptable for children at school, although problems with this technology were reported frequently. Among the intended treatment goals for children, 69% of treatment goals reached the expected level or exceeded the expected level. In accordance with our results, Donna Thomas et al. [5] reported promising signs of treatment effectiveness for speech disorders (speech apraxia) in children using videoconferencing; so that all five children's speech improved significantly after the intervention. The results of a study by Choi et al. [24] demonstrated the effectiveness and feasibility of the remote speech therapy program for patients with chronic aplasia. The mean scores of the Korean version of the western aphasia battery (K-WAB), compared to the initial scores, increased and the patients improved significantly.

In this regard, Bridgman et al. [14] confirmed the success and feasibility of the Lidcombe program (Child Behavioral Therapy) using web-based programs. The results of their study showed that the implementation of remote speech therapy using videoconferencing was as effective and economically feasible as its implementation in the clinic. The study by Agostini et al. [25] also showed the feasibility of telerehabilitation with regard to language functions. These researchers investigated the feasibility of remote rehabilitation in comparison with face-to-face treatment in patients with anomia (forgetfulness of names and difficulty in reminding or recalling words or names of things) after a stroke. They concluded that the face-to-face type of treatment had no significant effect on treatment. In line with the present study, Carey et al. [11] evaluated the effects of the Camperdown Therapy Program on stuttering adolescents using webcam. The results indicated significant improvement in stuttering of participants who received the treatment services through videoconferencing.

Along with this study, Susan Grogan-Johnson et al. [26] introduced live video conferencing communication as a practical way to provide remote health services to children with speech disorders in rural schools where speech-language pathologists were inaccecible. They concluded that 98% of students had a significant improvement in their voice and speech after using telemedicine. The results of Friedler's study [4] also showed that remote rehabilitation was not only feasible and suitable for the treatment of language-related patients, but also as effective as face-to-face treatment. The results of the study by Waite et al. [27] also confirmed the effectiveness of Internet-based remote health systems for assessing childhood language disorders. Participants in Johnson et al.'s study [28] strongly supported the treatment provided through videoconferencing and believed that videoconferencing was a promising way to render speech-language health services to schoolchildren. Crutchley and Campbell [29] stated that the distant speech-language treatment at a school in a remote North Carolina village was a satisfactory service model and suggested it to other schools in the district. In conjunction with the present study, the findings of a research showed that interactive video conferencing could provide a practical and effective care delivery model. In this study, the stuttering severity of all patients decreased after the treatment [3]. Although various studies provided information regarding the feasibility and effectiveness of tele-health, there are some drawbacks regarding this service. Two survey studies reported that the technical difficulties were one of the challenges therapists faced when providing this service [8, 30]. In a qualitative study, participants reported challenges including less patient engagement, difficulty regarding sharing information within the care team, and greater inefficiency when providing mental health [31]. Another study [32] negative experiences and challenges of participants consisted of low-quality of technology infrastructure for tele-speech therapy including low quality of shared images and videos, ineffective communication, insufficient sympathy, indirect communication, and technology incompetency.

The application of GAS in measuring the improvement of the stuttering problem was the strength of this study. Given the fact that patients are involved in defining and measuring this scale, it increases the patients' incentive to collaborate and participate in the treatment process. The effectiveness of this tool was confirmed in other studies regarding the rehabilitation interventions, which made it an appropriate scale in examining the treatment outcomes after interventions [33]. However, no study has ever used GAS in Iran. In comparison with the previous studies [6, 7] that used GAS, the number of patients and the number of treatment goals were higher in the present study. Moreover, participants of various age groups (14–40 years) were investigated in our research, which is different from previous studies.

One of the limitations of this study is the lack of evaluation of treatment stability. As stability in treatment of stuttering problem plays an important role in patients speech, it is required to measure the maintenance phase of the proposed intervention. Moreover, we provided the treatment through Skype; this may affect the results of the study. Since a tailored and specific platform suitable for speech therapy may create more confidence in therapists and patients and lead to better results. This is also recommened on other study providing tele-monitoring [34].

Since this study showed the effectiveness of tele-rehabilitation, it is recommended that the Ministry of Health and Health Policymakers provide the necessary infrastructure for the successful implementation of a remote speech therapy system. The successful launch of such a system requires further research on challenges, infrastructure, barriers, and other dimensions of the implementation of such a system.

## Conclusion

More than half of the patients with stuttering achieved their therapeutic goals in the current study. Therefore, rehabilitation of speech-language disorders using videoconferencing was effective for the participants.Re In other words, the patients' stuttering severity decreased significantly after the intervention. Since the lack of treatment follow-up caused by the inaccessibility of speech-language pathologists in all areas, providing tele-rehabilitation can improve the patient's conditions. In this regard, the healthcare organizations, in cooperation with the Ministry of Communications and Information Technology, are recommended to provide the required facilities and infrastructures to launch videoconference rehabilitation for people who do not have appropriate access to speech therapies.

## Data Availability

The data generated and analyzed during this study are available from the corresponding author on reasonable request.

## Abbreviations

GAS: Goal attainment scaling
SS%: Percentage of stuttered syllables
K-WAB: Korean version of the western aphasia battery
